# The Effects of Statins on Neurotransmission and Their Neuroprotective Role in Neurological and Psychiatric Disorders

**DOI:** 10.3390/molecules26102838

**Published:** 2021-05-11

**Authors:** Michał Kosowski, Joanna Smolarczyk-Kosowska, Marcin Hachuła, Mateusz Maligłówka, Marcin Basiak, Grzegorz Machnik, Robert Pudlo, Bogusław Okopień

**Affiliations:** 1 Department of Internal Medicine and Clinical Pharmacology, Medical University of Silesia, Medyków 18, 40-752 Katowice, Poland; d200998@365.sum.edu.pl (M.H.); mmaliglowka@sum.edu.pl (M.M.); mbasiak@sum.edu.pl (M.B.); gmachnik@sum.edu.pl (G.M.); bokopien@sum.edu.pl (B.O.); 2 Department of Psychiatry, Faculty of Medical Sciences in Zabrze, Medical University of Silesia, 40-055 Katowice, Poland; joanna.smolarczyk@med.sum.edu.pl (J.S.-K.); rpudlo@sum.edu.pl (R.P.)

**Keywords:** dopamine, acetylcholine, glutamate, BDNF, serotonin, neurotransmitters, statins, neurodegenerative diseases, stroke, depression

## Abstract

Statins are among the most widely used drug classes in the world. Apart from their basic mechanism of action, which is lowering cholesterol levels, many pleiotropic effects have been described so far, such as anti-inflammatory and antiatherosclerotic effects. A growing number of scientific reports have proven that these drugs have a beneficial effect on the functioning of the nervous system. The first reports proving that lipid-lowering therapy can influence the development of neurological and psychiatric diseases appeared in the 1990s. Despite numerous studies about the mechanisms by which statins may affect the functioning of the central nervous system (CNS), there are still no clear data explaining this effect. Most studies have focused on the metabolic effects of this group of drugs, however authors have also described the pleiotropic effects of statins, pointing to their probable impact on the neurotransmitter system and neuroprotective effects. The aim of this paper was to review the literature describing the impacts of statins on dopamine, serotonin, acetylcholine, and glutamate neurotransmission, as well as their neuroprotective role. This paper focuses on the mechanisms by which statins affect neurotransmission, as well as on their impacts on neurological and psychiatric diseases such as Parkinson’s disease (PD), Alzheimer’s disease (AD), vascular dementia (VD), stroke, and depression. The pleiotropic effects of statin usage could potentially open floodgates for research in these treatment domains, catching the attention of researchers and clinicians across the globe.

## 1. Introduction

Statins are the most widespread group of lipid-lowering drugs in the world [1]. For this reason, they are recommended for the primary and secondary prevention of cardiovascular events [2]. For many years, other effects of this group of drugs have been well known, which are primarily focused on anti-inflammatory activity [3,4]. The first scientific reports on the impacts of antilipid therapy on psychiatric and neurological diseases appeared in the 1990s. In 1990, Muldoon et al. proved that cholesterol-lowering therapy increases the risk of death in men as a result of accidents and suicide [5]. Subsequent reports also showed a relationship between cholesterol levels and the occurrence of anxiety, depression, and related suicide [6,7]. Moreover, despite very ambiguous results concerning these effects, meta-analyses have shown that statins reduce depressive symptoms and the frequency of hospitalization caused by intensification of these symptoms [8,9]. At the same time, reports began to appear in which researchers described the relationship between cholesterol level and the symptom severity in neurodegenerative diseases such as Alzheimer’s disease (AD) and Parkinson’s disease (PD) [10,11]. These observations prompted researchers to look for a potential mechanism of action by which statins act on neurotransmitter systems to influence neurological and psychiatric disorders. 

This study aims to systematize the current knowledge about the potential mechanisms by which statins affect cholinergic, dopaminergic, glutaminergic, and serotonergic transmission, as well as the impact of these interactions on the development and progression of neurodegenerative diseases and psychiatric disorders. Obviously, the effects of statins on neurological diseases through their lowering of the amount of total cholesterol and antiatherosclerotic effects seem not to be overlooked. However, in our manuscript, we only focus on their effects on neurotransmission and their neuroprotective role, because this topic is still a subject of discussion among scientists and requires further clinical research [12,13]. In this publication, we try to systematically review the current scientific data from international reports. For this purpose, the PubMed databases were reviewed in order to isolate reports according to the following key phrases: “statins and neurotransmission”, “lipid signaling and neurotransmission”, “statins and neurodegenerative diseases”, and “statins and psychiatric disorders”.

## 2. Statins–Structure and Permeability

Statins are drugs whose primary mechanism of action is to inhibit 3-hydroxy-3-methylglutaryl coenzyme A reductase (HMGCR). This is related to the ability of the pharmacophore, which for all statins is a dihydroheptanoic acid, to lower HMGCR activity. However, it is not a pharmacophore but rather the covalently related hydrophobic ring system that determines the chemical properties of individual statins, such as their solubility or pharmacokinetic properties.

Statins are divided into two categories: type 1, natural or semi-synthetic (these include lovastatin, simvastatin, and pravastatin); and type 2, otherwise known as fully synthetic [14]. One of the differences between the types of statins is their ability to bind to HMGCR. Type 2 statins, such as atorvastatin and rosuvastatin, are able to interact more strongly with HMGCR due to their greater hydrogen binding capacity [15]. The second difference is their different hydrophilicity. Lovastatin, simvastatin, fluvastatin, pitavastatin, and cerivastatin are more lipophilic, while rosuvastatin and pravastatin are more hydrophilic. This feature is very important in the context of the pleiotropic effects of this group of drugs. Lipophilic statins have a greater ability to passively pass from blood to tissues, including the ability to cross the blood–brain barrier (BBB). This results, among other things, in a greater severity of side effects. Hydrophilic statins, due to the necessity to penetrate inside the cells by active transport, show a more hepatoselective effect, which means that other effects, apart from lipid-lowering activity, are less intense. Recent studies confirm this difference in the ability to produce pleiotropic effects after taking type 1 and type 2 statins [16].

In summary, the basic differences between the two types of statins consist of their differences in chemical structure, which result in different pharmacokinetics for both types and the ability to penetrate into different types of tissues. Thereby, this results in the differentially expressed capacity to induce pleiotropic effects by different types of statins, including actions on the central nervous system (CNS).

## 3. Statins and Dopaminergic Neurotransmission

### 3.1. Structure and Synthesis of Dopamine

**Figure 1:**
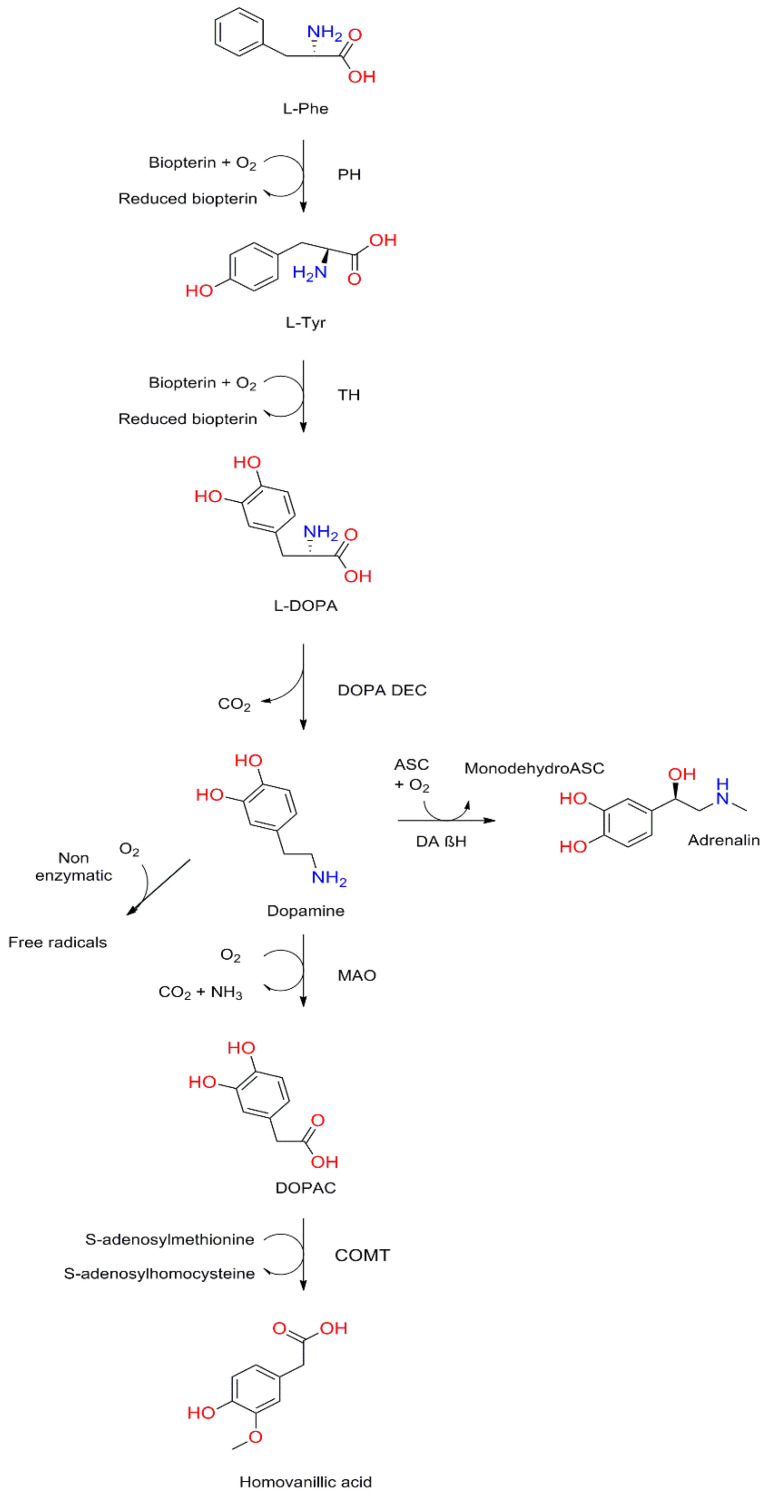
Dopamine synthesis and degradation pathways. L-Phe, L-phenylalanine; PH, phenylalanine hydroxylase; L-Tyr, L-tyrosine; TH, tyrosine hydroxylase; L-DOPA, levo-dopa; DOPA DEC, L-DOPA decarboxylase; ASC, ascorbic acid; DA βH, dopamine β-hydroxylase; MAO, monoamine oxidase; DOPAC, 3,4-dihydroxyphenylacetic acid; COMT, catechol-o-methyltransferase.

The chemical 4-(2-aminoethyl)-1,2-benzenediol, known as dopamine (DA), is one of the most important neurotransmitters in the human nervous system. It is synthesized from phenylalanine (Phe), which is converted by phenylalanine hydroxylase (PH) to tyrosine (Tyr), which is a precursor of several important bioactive molecules. Two enzymes are involved in the conversion of Tyr to DA: L-tyrosine hydroxylase (TH), used as a marker for dopamine-producing cells, and levo-dopa decarboxylase (DOPA DEC). DA synthesized in cells can be used and is then degraded, but in some cells with dopamine beta-hydroxylase (DAβH), such as adrenal gland cells, it takes part in the synthesis of norepinephrine (NA). The detailed synthesis and degradation process is shown in Figure 1. 

In the CNS, the process described above is carried out by groups of neurons called dopaminergic neurons, which can be found in many different parts of the CNS but are mostly concentrated in the substantia nigra pars compacta (SNpc). These neurons are responsible for receiving signals traveling from the striatum, then processing them and further transmitting them to other parts of the CNS, such as the globus pallidus (GP), thalamus, or substantia nigra pars reticulata (SNpr). DA, through the signaling pathways described above, participates in many processes regulated by the CNS, from the control of motor functions to cognition. Its action is based on two well-known mechanisms. The first one, called wiring transmission, involves the release of DA by neurons into the synaptic cleft, which then the released neurotransmitter acts on receptors in the postsynaptic membrane. The second mechanism, which is much more interesting, is called volume transmission, in which DA released from the presynaptic membrane reaches the extracellular space and binds to the dopaminergic receptors of neurons, which are not in direct contact with the cell from which it is released [17,18,19].

### 3.2. Dopamine Receptors

**Figure 2:**
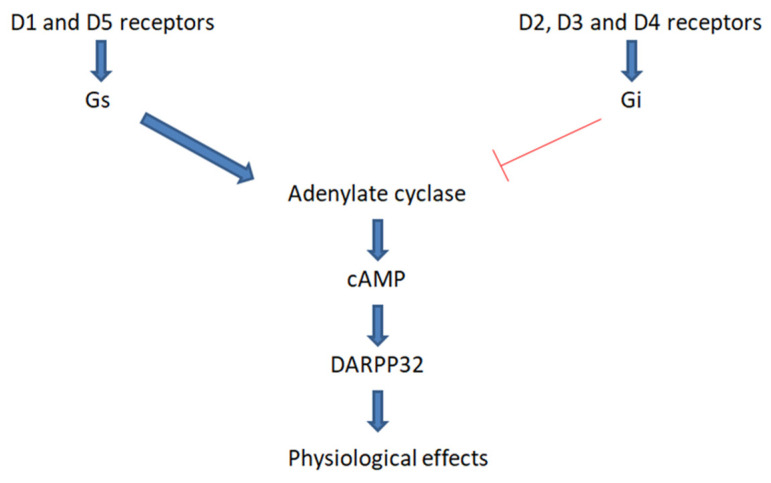
Mechanism of action of dopaminergic receptors. The binding of dopamine to the D1 and D5 receptors causes the activation of adenylate cyclase (AC) via the Gs protein. Activation of AC causes an increase in the concentration of cyclic adenosine monophosphate (cAMP), which results in an increase in the concentration of DA- and cAMP-regulated phosphoprotein of 32 kDa molecular weight (DARPP32), which penetrates into the cell nucleus, inducing a physiological response of the cell to dopamine. The reverse reaction is caused by the binding of dopamine to the D2, D3, and D4 receptors, which causes the inhibition of AC through the Gi protein.

So far, five dopamine receptors (D1, D2, D3, D4, and D5) have been described. They belong to the G-protein-coupled receptor (GPCR) family. It is considered that the binding of DA to these receptors leads to changes in the concentration of cyclic adenosine monophosphate (cAMP), which changes the activity of kinase DA- and cAMP-regulated phosphoprotein of 32 kDa molecular weight (DARPP32), which is a key protein in dopaminergic neurotransmission. This is mediated by G proteins associated with the individual dopaminergic receptors. The Gs protein, associated with D1 and D5 receptors, causes the activation of adenylate cyclase (AC), which causes an increase in cAMP concentration, while the Gi protein, associated with D2, D3, and D4 receptors, causes inactivation of AC and a decrease in cAMP concentration [20]. This process is shown in Figure 2. Importantly, dopamine receptors can be found not only in the brain, but also in other types of tissues, which leads to the conclusion that DA is more than just a neurotransmitter [21,22,23]. 

Researchers have repeatedly described the presence of different variants of dopamine receptors and many polymorphisms of the genes encoding these receptors. Importantly, some of these polymorphisms may be associated with some types of addiction, such as alcohol or drug addiction [24,25,26,27]. The variety of DA’s effects and the variety of drugs affecting dopaminergic transmission come from the ability of dopamine receptors to form complexes in which they combine with each other or with other types of membrane receptors. Importantly, each of the heteromers formed in this way transmits a different signal inside the cell after activation by DA, so each has a different physiological role and pharmacological properties [28,29]. Examples of such heteromers are homeotropic heteromers D1–D3 [30], D2–D3 [31], D2–D5 [32], and D2–D4 [33] and heterotropic heteromers A1–D1 [34], A2A–D2 [35], D1–H3 [36], D2–H3 [37] and D4-adrenergic [38]. The presence of these heteromers is important not only in physiological mechanisms, such as the regulation of melatonin production by the pineal gland [39], but also in the pathogenesis of diseases such as PD. One of the main causes of this disease is the antagonism between dopaminergic transmission and purinergic regulation of neurotransmitter release caused by the presence of A1–D1 and A2A–D2 heteromers [40,41]. 

### 3.3. Cholesterol and Dopaminergic Transmission

Because disorders of dopaminergic transmission were found to be among the main causes of PD development, researchers have also described other mechanisms that are responsible for such disorders. One of the described mechanisms is a disorder of DA release and reuptake regulated by the dopamine transporter (DAT) and vesicular monoamine transporter 2 (VMAT2) proteins. These proteins are key regulators of DA release into the synaptic cleft. Because the structure of DAT consists of two conserved cholesterol-like molecules, it is suggested that the protein may interact directly with cholesterol. In the absence of cholesterol, changes occur in the conformation of this protein that enhance DA reuptake, and in the presence of bound cholesterol these conformational changes are inhibited [42]. Moreover, cholesterol strengthens H-bonds, which bind DA and levo-dopa (L-DOPA) to the cell membrane, influencing their metabolism [43]. It is worth noting that the relationship between cholesterol and DA is not one-sided. Excess DA is responsible for the increase in cholesterol synthesis by activating the c-Jun N-terminal kinase (JNK3)/sterol regulatory element-binding protein 2 (SREBP2) signaling pathway in astrocyte colonies [44]. 

Another described mechanism by which cholesterol levels may influence the development of PD is an increased concentration of oxysterols produced from cholesterol. Evidence from studies shows that an elevated concentration of 24-hydroxycholesterol (24-OHC) in the cerebrospinal fluid of patients suffering from PD correlates with the worst prognosis [45]. Accordingly, it has been proposed that 24-OHC becomes a biomarker in PD. Other studies also indicate the effect of 27-hydroxycholesterol (27-OHC), another oxysterol. In dopaminergic neurons, this causes an increase in α-synuclein concentration by inhibiting proteasomes and activating the liver X receptors (LXRs) [46,47]. Moreover, 27-OHC induces inhibition of the estrogen receptor, which leads to inhibition of the expression of TH, and thus slows down the synthesis of DA [48].

**Figure 3:**
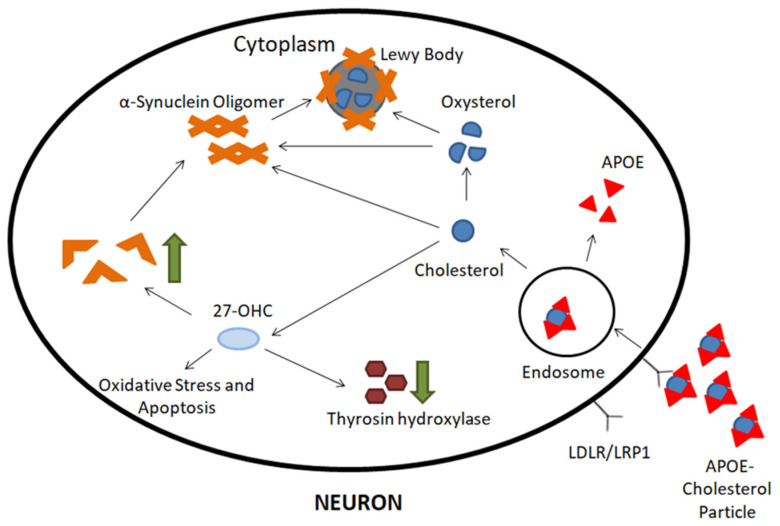
Cholesterol metabolism in Parkinson’s disease. After endocytosis of apolipoprotein E (APOE)-cholesterol particles, cholesterol is metabolized to 27-hydroxylcholesterol (27-OHC) and other oxysterols. Furthermore, 27-OHC can increase α-synuclein synthesis, downregulate tyrosine hydroxylase (TH) activity, and cause oxidative stress and apoptosis. In addition, excessive cholesterol and oxysterol can promote α-synuclein aggregation, and aggregated α-synuclein will eventually form Lewy bodies (LBs).

The last mechanism by which cholesterol metabolism may affect neurodegenerative processes within dopaminergic neurons is related to the relationship between cholesterol and accumulated α-synuclein deposits [49]; α-synuclein is a protein whose overexpression may inhibit the transport and release of neurotransmitters from synaptic vesicles [50]. The α-synuclein molecule is made up of 140 amino acids and can be broken down into three domains: the *N*-terminal lipid-binding α-helix, the amyloid-binding central domain (known as NAC), and the *C*-terminal acidic tail. Importantly, its structure is characterized by a tandem repeat in the α-helix similar to those found in apolipoproteins. It follows that this protein has a structure similar to apolipoproteins [51,52]. The two cholesterol binding domains thus give the α-synuclein molecule a strong tendency to bind to lipid membranes, especially in cholesterol-rich regions. Moreover, studies conducted in vitro and in animal models show that α-synuclein could play a role in cholesterol transport [53,54,55]. Studies have reported that cholesterol may affect the interaction between α-synucelin oligomers and the cell membrane, which leads to membrane destruction, and thus cell death [56]. Moreover, with a low concentration of apolipoprotein E (APOE), α-synuclein is more prone to aggregation, which suggests that these two proteins may be competitively bound to cholesterol [57]. The mechanisms described above are illustrated in Figure 3.

It is important to emphasize that the last two described mechanisms concerning oxysterols and the deposition of α-synuclein are responsible for neurodegeneration not only within dopaminergic neurons, but also within other types of neurons, which may result in the occurrence of diseases such as AD [58,59] or Lewy body dementia (LBD) [60].

### 3.4. Influence of Statins on Dopaminergic Transmission

Due to the above-described mechanisms involving the influence of cholesterol on neurodegenerative processes and dopaminergic transmission, researchers’ attention has been drawn to the influence of lipid-lowering therapy with statins on the course of neurodegenerative diseases such as PD and AD. Studies show that chronic statin treatment exerts an anti-inflammatory effect, inhibits oxidative stress, and has a preventive effect on apoptosis of neurons, including dopaminergic neurons [61,62]. This effect is mainly focused on inhibiting the release of pro-inflammatory cytokines and the activation of nuclear factor kappa-light-chain-enhancer of activated B (NF-κB) cells [61]. It has also been proven in cell models that simvastatin, by inhibiting *N*-methyl-*D*-aspartate receptor 1 (NMDAR1), inhibits the inflammatory process within nerve cells [63]. Another mechanism by which statins inhibit neurodegenerative processes is in vitro reduction of beta-amyloid (Aβ) concentration in nerve cells [64], as well as activation of a disintegrin and metalloproteinase domain-containing protein 10 (ADAM10) and increased activity of phospholipid transporter (PLTP), which reduces the concentration of plasma-phosphorylated tau181 (p-tau181) [65]. So far, however, there are no reports describing the influence of statins on the process of dopaminergic transmission by modifying cholesterol levels. All preclinical effects of statins on the process of neurotransmission and neuroprotection discussed in this article are summarized in the Table S1.

In connection with the above-described mechanisms, many clinical trials have been conducted to determine the effects of lipid-lowering therapy on the course of PD and AD. In the case of AD, previous studies have shown that statin therapy reduces the risk of AD by up to 70% [66,67]. However, later studies showed no correlation between this therapy and the risk of dementia [68]. These differences may be caused not only by differences in disease severity between patients, but also by the different chemical properties of the statins. For example, lipophilic statins, due to the ease of crossing BBB, show a stronger effect than hydrophilic ones in inhibiting the progression of AD [69]. In the case of PD research, the divergence is even greater. According to a meta-analysis prepared by Sheng et al., most observational studies show that statins can reduce the risk of PD by up to 26% [70], while several clinical studies have shown that statins are harmful to patients suffering from PD. Studies on the efficacy of statins for the prevention of PD and AD are summarized in Table 1.

Because of these uncertainties regarding the research on groups of patients with PD and AD, well-designed controlled trials are needed to clearly demonstrate the effects of these groups of drugs on neurodegenerative diseases.

## 4. Statins and Cholinergic Neurotransmission

### 4.1. Cholinergic Transmission in Pathogenesis of Vascular Dementia

Vascular dementia (VD) is the second most frequent subtype of cognitive disorders after AD [80]. Chronic cerebral hypoperfusion (CCH), the crucial factor, which is caused by negative modification of cerebral blood vessels and associated with the initiation and progression of VD, results in numerous molecular changes inside the brain cells and neuronal junctions, including neurotransmitter and lipid metabolism disturbance, mitochondrial dysfunction, alteration of growth factors, neuroinflammation, and overproduction of reactive oxygen species (ROS) [81].

Acetylcholine (ACh) plays an important role in the physiological functioning of the CNS. The neuronal synthesis of Ach from choline and acetyl-CoA is catalyzed by acetylcholine transferase enzyme (ChAT). Subsequently, Ach, transported in vesicles with the involvement of vesicular acetylcholine transporter (VAChT), is released into the synaptic cleft, where it can bind to receptors. Within the synapse, ACh is degraded by acetylcholinesterase (AChE), resulting in the formation of acetic acid and choline, a precursor for the synthesis of new ACh [82,83]. 

There are two types of ACh receptors: metabotropic muscarinic receptors (mAChRs) and ionotropic nicotinic receptors (nAChRs). The family of mACHRs contains five subtypes of GPCR, M1–M5. The larger group, with pentameric nAChRs made up of α and β subunits, contains nonselective cation channels. The effects of binding ACh to cholinergic receptors can result in stimulation or inhibition of neuronal signaling, depending on the receptor subtype and its location on a pre- or postsynaptic membrane [84,85,86].

The basal forebrain cholinergic system, comprising the medial septal nucleus, the nucleus of the diagonal band of Broca, and the nucleus basalis of Meynert, is widely accepted as a crucial structure of cognitive functions. It is involved in the regulation of memory, attention, and emotions [87]. There is some evidence that cholinergic mechanisms are also responsible for the control of cerebral blood flow [88,89]. This may partially explain the pathogenesis of VD and deterioration in the course of disease. The ongoing neuroinflammation in patients with VD may also be attenuated by activation of the cholinergic system (α7 nAChRs) [90].

Ischemic lesions observed in various areas of the brain in patients with VD can cause decreased amounts of ACh, gamma-aminobutyric acid (GABA), or DA [81]. The most profound deficits of common cholinergic markers, such as ChAT, AChE, and VAChT, appear in the temporal cortex and hippocampus [91]. However, the latest research suggests that more evident loss of cholinergic function occurs in the brains of patients with mixed dementia [92]. A decreased Ach level is also observed in cerebrospinal fluid [93,94].Findings concerning changes in cholinergic receptor numbers are contradictory for mAChRs [95,96]. The amount of nAChRs seems to be preserved in VD [97]. The cholinergic reductions observed in the course of VD may be responsible for the cognitive impairment [98].

### 4.2. Influence of Statins on Cholinergic Transmission

Statins, due to their pluripotential pleiotropic effects on brain cells and vessels beyond lipid-lowering actions, have been widely tested as drugs for the treatment of VD [99]. In L-methionine-induced VD, the use of simvastatin ameliorated behavioral status and increased the amount of ACh in the brain tissue of rats [100]. These encouraging observations have not been seen in human patients with VD. Moreover, some studies indicated potential harmful effects of statin therapy on neuropsychological tests of attention and psychomotor speed [101]. Recent assessments of randomized, placebo-controlled trials did not confirm the clinical significance of these observations [102]. Although statin therapy is useful in primary and secondary prevention of vascular incidents, including strokes, to date there is no conclusive proof that statins have a major influence on the prevention, incidence, or progression of VD [80,103].

## 5. Statins and Glutamatergic Neurotransmission

### 5.1. Structure and Synthesis of Glutamate

Glutamate (Glu), the anion of glutamic acid, acts as a neurotransmitter. It is the major excitatory transmitter within the human nervous system, accounting for over 85% of the synaptic connections in the CNS. Glu can be produced de novo from α-ketoglutaric acid as part of the citric acid cycle. In CNS, Glu is synthesized in the glutamate–glutamine cycling mechanism. These reactions occur in presynaptic neurons or glial cells. Glu is transported within presynaptic neurons by vesicular glutamate transporters and then released into the synaptic cleft. Inside the synaptic cleft, anions of glutamic acid can bind several different postsynaptic receptor types, named according to their agonists: kainite receptor (KAR), α-amino-3-hydroxy-5-methyl4-isoxazole propionic acid receptor (AMPAR), and N-methyl-D-aspartate receptor (NMDAR). Glu binds to these receptors with different affinity and induces differential effects on target postsynaptic neurons [104,105]. For this part of the review, we would like to focus on NMDARs.

### 5.2. N-Methyl-D-Aspartate Receptor

Belonging to the neurotransmitter receptors, NMDARs constitute the largest subclass of glutamate-gated ion channels in human excitatory synapses, which have a main part in neuroplasticity, neuronal development, and learning and memory processes [106]. NMDARs are heteromeric molecules formed of one obligatory GluN1 (also referred to asNR1) incorporated with various constellations of GluN2 (also named NR2) and GluN3 subunits, which take several variants: the single GluN1 subunit with eight isoforms, four GluN2 subunits (GluN2A–GluN2D), and two GluN3 subunits. Both the GluN1 and GluN2 subunits participate in the development of the NMDAR ion channel. Each NMDAR has a similar membrane subunit topology, which is dominated by a large extracellular N-terminus, a membrane region containing three transmembrane segments, a re-entrant loop, and an extracellular loop between the transmembrane segments. Intracellularly, it is situated in a carboxyl (C) domain of various sizes, and miscellaneous proteins interact in this site [107,108,109,110,111].

NMDAR is extraordinary in that the opening of the channel requires the merging of two different agonists, Glu and glycine (Gly). Glu binds to the GluN2 subunit, while the binding site for Gly, the co-agonist, is located on the GluN1 subunits. The NMDAR ion channel is permeable to monovalent cations, such as Na^+^ and K^+^, and divalent cations, especially Ca^2+^. It is regulated by voltage-dependent Mg^2+^ blockade. Accordingly, both depolarization of the postsynaptic neurons and presynaptic release of Glu is needed for maximal current flow through the NMDAR channel. The concentration of Gly in most synapses is usually enough to allow for efficient NMDAR activation [108,110,111,112,113]. NMDAR is mainly located at dendritic spines, where through specific interactions it connects to intracellular molecules of the postsynaptic multiprotein network known as the postsynaptic density (PSD); for the subunit GluN1thisis neurofilament light protein (NF-L), while for GluN2 these are PSD-95, PSD-93, and synapse-associated protein 102(SAP102). In addition to their function as PSD cytoskeleton proteins, PSD-95 and SAP102 are involved in transporting newly synthesized NMDA receptors to the PSD. Build or behavior irregularities for these molecules could disturb receptor signaling, interfere with NMDAR trafficking, and finally affect neurotransmission [107]. The number of NMDARs can be modified, which contributes to the mechanism regulating synaptic efficacy and their remodeling [114]. With disorder in the NMDA signal pathway, glutamatergic transmission could exacerbate brain diseases, including psychiatric, neurodegenerative, and excitotoxic disorders [112].

### 5.3. Role of Glutamatergic Transmission in the Pathogenesis of Stroke

Excitotoxicity is a pathological process that causes cell death as the result of the toxic actions of excitatory amino acids. Considering that Glu is the main excitatory neurotransmitter in the human CNS, excitotoxicity typically refers to the trauma and death of neurons that occur from prolonged exposition to Glu. It comes from overloading the cell with ions, mainly calcium, which is notably neurotoxic and leads to the activation of enzymes that degrade proteins, nucleic acids, and other components of the cell. It is considered that Ca^2+^ inflow through NMDA channels is a common pathway of neuronal cell death. Excess levels of Glu in the CNS are associated with increased intracellular calcium ions levels, which cause a rise in their concentration in sensitive organelles such as mitochondria and the endoplasmic reticulum (ER) [115]. The mitochondrial uptake of calcium results in the production of ROS [116].

Stroke is a major cause of death, causing approximately 9% of deaths worldwide. Up to 80% of the global burden of stroke is attributed to ischemic stroke. This is a type of stroke characterized by a temporary or permanent reduction in blood perfusion due to embolic or thrombotic occlusion in cerebral arteries. Most cases of focal ischemia result from occlusion of the middle cerebral artery [117]. There is evidence that stroke leads to the release of large amounts of Glu, which activates NMDARs, and that glutamate-induced excitotoxicity participates in the neuronal death observed after stroke [118]. The first step of excitotoxicity during acute ischemia is a sudden increase of Glu levels in the ischemic region of the brain. Activation of NMDARs does not always lead to excitotoxicity. There is evidence that this receptor has dual effects, depending on the subunit subpopulation. GluN2A tends to promote neuronal survival and protects the brain against excitotoxic injury, whereas the GluN2B subunit promotes neuronal death. Cerebral ischemia triggering excessive activation of NMDARs induces rapid and specific upregulation of GluN2B [119].

Previous studies found that the excitotoxic process connected with acute ischemia is responsible for redistributed microtubule-associated proteins (MAP2) and loss of microtubule stability as a consequence. Normally these proteins are engaged in the regulation of vesicle transport during the creation or recovery of neuronal pathways [120]. Complexes of cadherin or catenins and actin are involved in maintaining the structure of the scaffolding proteins. Cerebral ischemia leads to structural damage of the cytoskeleton mediated by RhoGTPasas imbalance, Ras homolog family member A (RhoA) activation, and inactivation of Ras-related C3 botulinum toxin substrate (Rac), related to the rupture of adhesion. A study by Cespedes-Rubio showed that RhoA activity is increased in cell death processes due to excitotoxicity [121]. The inflammatory response induced by ischemia triggers the activation of signaling pathways, finally leading to neuronal cell death. There is evidence confirming that the phosphatidylinositol 3-kinase (PI3K)-protein kinase B (Akt) signaling pathway is one of the serious signaling paths taking part in neuronal apoptosis. Glycogen synthase kinase-3β (GSK-3β) is an important protein downstream of Akt. Sustained activation of GSK-3β is pro-apoptotic in cerebral ischemia because it leads to hyperphosphorylation of tau, with consequent microtubule destabilization [122].

### 5.4. Influence of Statins on Glutamatergic Transmission and Their Neuroprotective Effect

Researchers continue to look for new effects of statin treatment in stroke, in primary and secondary prevention and in the acute phase of ischemia. Statins exert protective effects in vivo and in experimental models of stroke. Recent meta-analyses showed that statin therapy significantly reduces the overall risk and mortality rate of stroke, in both primary and secondary prevention, which confirms that accurate control of the lipid profile is needed [123,124]. Beyond their effects on the lipid profile, statins are also credited with pleiotropic effects. Among the pleiotropic effects reported in cerebral ischemia is improved endothelial function, stabilized atherosclerotic plaque, impaired inflammation with a concomitant decrease in ROS, and inhibition of the thrombogenic response [125]. Increasingly, studies are examining the effects of statin treatment on NMDARs and the process of excitotoxicity after acute ischemia. The precise mechanisms involved in these actions are not completely known. Studies indicate that NMDA channels are involved in the neuroprotective mechanism induced by statins to promote neuronal recovery after cerebral focal ischemia. 

Gutierrez-Vargas et al. examined the influence of a high dose of atorvastatin on NMDA receptors after cerebral ischemia in laboratory rats. This work suggests that atorvastatin protects neurons after ischemia, restoring the balance of subunits by decreasing GluN2B upregulation [106]. Additionally, the same study described that treatment with atorvastatin improves the adhesion protein complex of NMDARs associated with PSD-95, influences Akt activation in promoting cell survival, and in turn promotes synaptic plasticity. Statins inhibit the synthesis of valid isoprenoids, such as farnesyl pyrophosphate (FPP) and geranylgeranyl pyrophosphate (GGPP), which are important intermediates for the post-translational modification of Rho GTPases, leading to the modulation of various cellular functions, e.g., decreased structural damage of the cytoskeleton [125]. Additionally, Gutierrez-Vargas et al. proved that atorvastatin used after ischemic stroke influences the recovery of the actin cytoskeleton and stabilizes microtubules by increased activity of Rac and RhoA reduction [126]. Another mechanism of neuroprotection by statins involves their influence on inflammation through a number of proinflammatory cytokines. Tuttolomondo et al., in the first human randomized trial, proved that early administration of high-dose atorvastatin caused a significantly lower serum level of inflammatory markers and may be related to a better prognosis after stroke [127]. Additionally, Campos-Martorell et al. showed that simvastatin used after acute ischemia had an influence on decreased oxidative stress [128]. Brain-derived neurotrophic factor (BDNF) induces neuronal proliferation and synaptogenesis and is also involved in the regulation of neurogenesis. After injury, it takes part in the recovery of neuronal tissue. Cerebral ischemia decreased levels of BDNF [129]. Atorvastatin used in the treatment of cerebral ischemia in animals led to recovered BDNF levels [106].

Considering that cerebral ischemia is one of the major global health problems with great costs for rehabilitation and recovery, more effective and accessible methods are needed to immediately reduce postischemic injury. Statins meet these criteria: they are cheap and easily available. Experimental models, experiments on rats, and preclinical studies have shown that they influence neuronal cells differently and could be used to reduce neurodegeneration after stroke. The above studies prove that large multi-center clinical studies are needed.

## 6. Statins and Serotoninergic Neurotransmission

### 6.1. Structure and Synthesis of Serotonin

**Figure 4:**
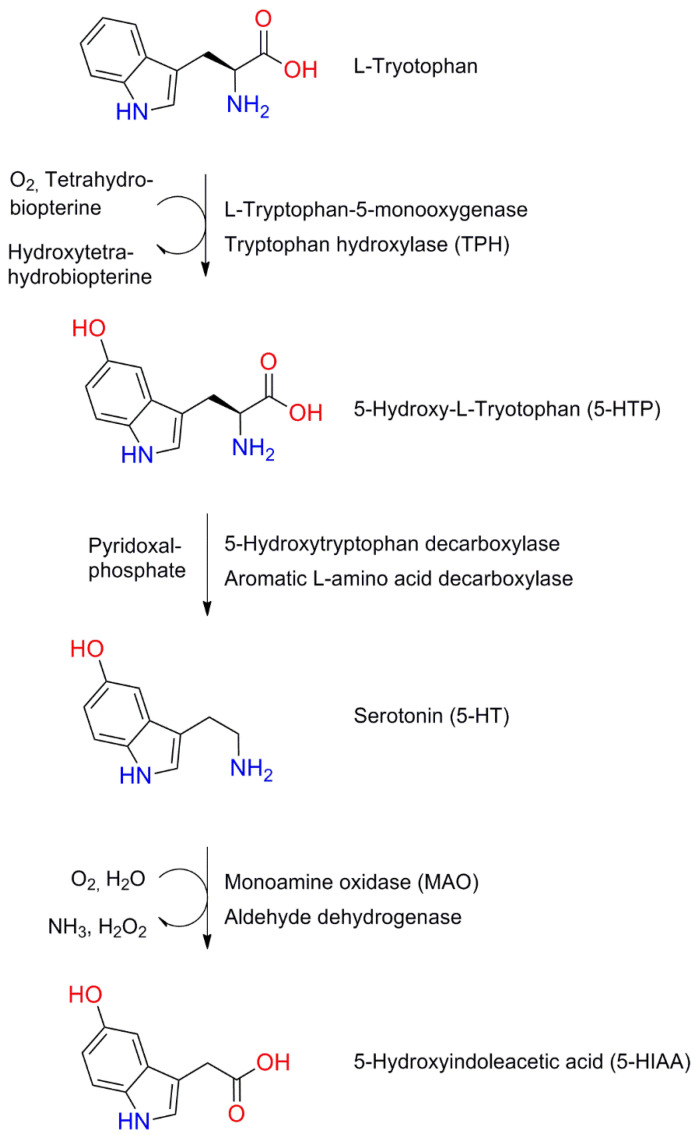
Serotonin synthesis and degradation pathways.

Serotonin (5-HT) is one of the oldest neurotransmitters; it is estimated that its receptors appeared 700–800 million years ago in unicellular eukaryotes, such as Paramecium caudatum [130]. It is a monoamine produced within both the CNS and the peripheral nervous system (PNS). In the CNS, serotonergic neurons can be found in the dorsalraphe nucleus (DRN) and median raphe nucleus (MRN) [131]. In the PNS, it is synthesized in the gastrointestinal (GI) system by gut neurons and enterochromaffin cells. The substrate for its production is tryptophan and the synthesis process follows the scheme shown in Figure 4 [132].

In the CNS, serotonergic neurons from DRN and MRN communicate with various areas within the cerebral cortex, limbic system, midbrain, and cerebellum [133]. Serotonin communication occurs mainly through volume transmission (VT) in the extracellular space and the cerebrospinal fluid (CSF). Serotonin travels from the source to target cells (neurons and astroglia) through energy gradients, leading to its diffusion and convection [134]. By interacting with its receptors, 5-HT is responsible for the regulation of many processes important for life, which include perception, mood, anxiety, aggression, cognitive functions, attention, sexual functions, and the circadian rhythm [131,135,136].

### 6.2. Serotonin Receptors and Transporters

Thirteen G-protein-coupled heptahelial serotonin receptors (5-HTRs) and one ligand-gated ion channel have been identified and are divided into seven distinct classes (5-HT_1–7_) [132,134]. All 5-HTRs are heteroreceptors associated with the postsynaptic membrane on nonserotonergic neurons. Presynaptically located autoreceptors (5-HT_1A,1B,1D_) respond to the regulation of 5-HT release through negative feedback and influence the neuronal firing rate. The 5-HTRs are located within the CNS, PNS, and other tissues, and the exact mechanisms of their action and the effects of stimulation are presented in Table 2 [132,137].

One of the new concepts of depression is that disturbances in integrated allosteric receptor–receptor interactions in highly sensitive 5-HT_1A_ heteroreceptor complexes may contribute to the induction of major depression (MD). For example, disruption or dysfunction in 5-HT_1A_-FGFR1 heteroreceptor complexes in the suture–hippocampal serotonin neuron systems may contribute to the development of MD [134].

Another important membrane protein involved in serotonergic transmission is the serotonin reuptake transporter (SERT). It is responsible for the removal of free 5-HT from the synaptic cleft, which directly affects the duration of 5-HTR activation. Some transporter-regulatory proteins, such as syntaxin 1A (Syn1A) and secretory carrier membrane protein 2 (SCAMP2), are involved in regulating the activity of SERT [138]. It is also known that some polymorphisms in the SERT gene are associated with the occurrence of depression, anxiety disorders, autism, and suicidality [139]; therefore, the process of 5-HT reuptake has become one of the most important points in therapy for depression disorders.

### 6.3. Influence of Statins on Serotoninergic Transmission

Due to the influence of statins on neurodegenerative diseases and cognitive disorders known from many studies, consideration was also given to their potential influence on psychiatric disorders. A possible mechanism of their action is to increase serotonin reuptake through the SERT receptor in a manner independent of the cholesterol synthesis pathway, as described in animal models [140]. The range of concentrations in which statins increase SERT uptake is wide and includes concentrations achieved in acute systemic treatment [140,141]. Such a mechanism would suggest a potential effect of intensifying or inducing depressive symptoms. However, a cohort study of the Swedish population published in 2020 suggested that the incidence of depressive disorders in the group of people taking statins was lower than in the general population [142].

Possible mechanisms underlying the antidepressant effects of statins may include anti-inflammatory, antioxidant, and lipid-lowering properties [143]. The potential anti-inflammatory effects of statins include lowering C-reactive protein (CRP) levels [144] andantioxidant activity [145], inhibiting the production of pro-inflammatory cytokines by monocytes [146], inhibiting lymphocytes by blocking the function of antigen-1 leukocytes (LFA-1) [147], and blocking T-cell activation [148]. The antidepressant mechanism of statins may also be related to their antiatherosclerotic effect and their influence on damage to small white matter vessels, which underlies the hypothesis of vascular depression [149]. Such injuries may predispose people to depression, accelerate its course, and reduce the effectiveness of antidepressants [143].

Despite the mechanisms described above and the retrospective studies conducted so far, the influence of statins on the incidence of depressive disorders is still unclear and requires further research.

## 7. Conclusions

To date, researchers have described a number of mechanisms by which cholesterol influences neuronal transmission. These mechanisms can also be influenced by statins, which has been confirmed in animal and cellular models. Additionally, many retrospective studies have described the beneficial effects of this group of drugs on neurological diseases and psychiatric disorders. So far, however, there have been no clinical trials that have unequivocally proven their beneficial effects on the diseases described in our paper. This opens up a wide field for researchers, especially as statins still remain one of the most widely used drug groups in the general population.

## Figures and Tables

**Table 1:** Studies on the efficacy of statins for prevention of Alzheimer’s disease (AD) and Parkinson’sdisease (PD).

Statins	Model	Group Size	Effects	References
All types	Rotterdam study	6992	Reduced risk of late-onset AD	Haag et al. [71]
	Prospective study	15,291	Incerased risk of PD	Huang et al. [72]
	Retrospective case–control analysis	2322	Lipophilic statins increased risk of PD and hydrophilic statins did not affect incidence of PD	Liu et al. [73]
	Population-based cohort study	232,877	Statins did not affect incidence of PD	Rozani et al. [74]
	Meta-analysis	3,845,303	Statins, especially atorvastatin, reduced risk of PD	Yan et al. [75]
	Meta-analysis	3,513,209	Decreased risk of PD	Bai et al. [76]
	Meta-analysis	2,787,249	Statins reduced risk of PD	Sheng et al. [70]
Atorvastatin	Randomized controlled trial	640	No therapeutic effect in AD	Feldman et al. [77]
	Randomized controlled trial	63	AD progressed slowly	Sparks et al. [78]
Lovastatin	Randomized controlled trial	160	Decreased serum Aβ	Friedhoff et al. [79]

**Table 2:** Serotonin (5-HT) receptor subtypes. CNS, central nervous system; cAMP, cyclic adenosine monophosphate; AC, adenylate cyclase; GIT, gastrointestinal tract; IP3, inositol-1,4,5-triphosphate; PKC, protein kinase C.

Receptor	Location	Mechanism of Action	Functions
5-HT_1A_	CNS	Decreased cAMP concentration by inhibition of AC	Learning and memory, depression, anxiety-like behaviors
5-HT_1B_	CNS, vascular smooth muscle	Decreased cAMP concentration by inhibition of AC	Aggression, antimigraine effects and vasoconstriction, depression and anxiety-like behaviors
5-HT_1C_	CNS, limfocytes	Not completely understood	Not completely understood
5-HT_1D_	CNS, vascular smooth muscle	Decreased cAMP concentration by inhibition of AC	Pain perception, antimigraine effects, and vasoconstriction
5-HT_1E_	CNS	Decreased cAMP concentration by inhibition of AC	Not completely understood
5-HT_1F_	CNS, uterus, heart, GIT	Decreased cAMP concentration by inhibition of AC	Pain perception, antimigraine effects, andanxiety-like behaviors
5-HT_2A_	CNS, PNS, thrombocytes, smooth muscles	Enhanced AC activity and IP3	Pain perception, sensorimotor, motivation, emotionalregulation, vasoconstriction, smooth muscles cell constriction, thrombocyte aggregation
5-HT_2B_	CNS, stomach	Enhanced PKC activity and IP3	Anxiety-like behaviors, smooth muscle cell constriction
5-HT_2C_	CNS, limfocytes	Enhanced PKC activity and IP3	Anxiogenesis, sexual behavior, pain perception, regulation of serotonergic neuron activity
5-HT_3_	CNS, PNS	Opening of Na^+^, Ca^2+^, and K^+^ channels, depolarization of plasma membrane	Vomiting reflex, anxiety-like behaviors
5-HT_4_	CNS, PNS	Increased cAMP concentration by activation of AC	Anxiety-like behaviors, learning and memory
5-HT_5A_	CNS	Decreased cAMP concentration by inhibition of AC	Learning and memory, emotional behaviors, acquisition of adaptive behavior, circadian rhythm
5-HT_6_	CNS, leukocytes	Increased cAMP concentration by activation of AC	Anxiety-like behaviors, learning and memory, cognition
5-HT_7_	CNS, GIT, vascular smooth muscles	Increased cAMP concentration by activation of AC	Regulation of sleep and circadian rhythm, thermoregulation, learning and memory, regulation of 5-HT release

## Data Availability

Not applicable.

## Supplementary Materials

The following are available online. Table S1: Preclinical effects of statins on neurotransmission and neuroprotection.
