# Supplementary material for: The Effects of Statins on Neurotransmission and Their Neuroprotective Role in Neurological and Psychiatric Disorders
Source: Molecules. 2021 May 11;26(10):2838. doi: 10.3390/molecules26102838 (PMC8150718; doi:10.3390/molecules26102838)
Supplement: Supplementary file 1 [file molecules-26-02838-s001.zip › molecules-1198533-SI.pdf]

Supplementary Information

The Effects of Statins on Neurotransmission and Their Neuroprotective Role in Neurological and Psychiatric Disorders

Michał Kosowski [1,*], Joanna Smolarczyk-Kosowska [2], Marcin Hachuła [1], Mateusz Maligłówka [1], Marcin Basiak [1], Grzegorz Machnik [1], Robert Pudlo [2] and Bogusław Okopień [1]

[1] Department of Internal Medicine and Clinical Pharmacology, Medical University of Silesia, Medyków 18, 40-752 Katowice, Poland

[2] Department of Psychiatry, Faculty of Medical Sciences in Zabrze, Medical University of Silesia, Katowice, Poland

Table S1. Preclinical effects of statins on neurotransmission and neuroprotection.

Statin	Effect	References
All statins	inhibition of nuclear factor kappa-light-chain-enhancer of activated B cells (NF-κB)	Sierra et al. [61]
	decreasing the structural damage of the cytoskeleton	Wang et al. [125]
	increasing serotonin reuptake by serotonin reuptake transporter (SERT)	Deveau et al. [140], Johnson-Anuna et al. [141]
	anti-oxidant activity	Shishehbor et al. [145]
	inhibition of lymphocytes by blocking the function of antigen-1 leukocytes (LFA-1)	Weitz-Schmidt et al. [147]
	blocking T-cells activation	Bu et al. [148]
Atorvastatin	decreasing GluN2B glutamate receptor upregulation	Gutierrez-Vargas et al. [106]
	improvement the adhesion protein complex N-methyl-D-aspartate receptor (NMDAR) associated with postsynaptic density protein 95 (PSD-95)	Gutierrez-Vargas et al. [106]
	influence of Akt kinase activation in promoting cell survival and in turn promote synaptic plasticity	Gutierrez-Vargas et al. [106]
	recovering of the actin cytoskeleton and stabilizes microtubules	Gutierrez-Vargas et al. [126]
	decreasing level of brain-derived neurotrophic factor (BDNF)	Gutierrez-Vargas et al. [106]

Simvastatin	inhibition of NMDAR1	Yan et al. [63]
	reduction of beta-amyloid (Aβ) concentration in nerve cells	Fassbender et al. [64]
	increasing the amount of acetylcholine (Ach) in brain tissue of rats	El-Dessouki et al. [100]
	decreasing oxidative stress	Campos-Martorell et al. [128]
	inhibition of the production of pro-inflammatory cytokines by monocytes	Ferro et al. [146]
Lovastatin	activation of a disintegrin and metalloproteinase domain-containing protein 10 (ADAM10)	Kojro et al. [65]
	increasing in the activity of the phospholipid transporter (PLTP)	Kojro et al. [65]
	reducing the concentration of plasmaphosphorylated tau181 (p-tau181)	Kojro et al. [65]
Pravastatin, simvastatin, and atorvastatin	lowering C-reactive protein (CRP) level	Jialal et al. [144]
